# High variability in the dosing of commonly used antibiotics revealed by a Europe-wide point prevalence study: implications for research and dissemination

**DOI:** 10.1186/s12887-015-0359-y

**Published:** 2015-04-16

**Authors:** Tuuli Metsvaht, Georgi Nellis, Heili Varendi, Anthony J Nunn, Susan Graham, Andre Rieutord, Thomas Storme, James McElnay, Hussain Mulla, Mark A Turner, Irja Lutsar

**Affiliations:** Institute of Microbiology, Tartu University, Tartu, Estonia; Clinic of Anaesthesiology and Intensive Care, Tartu University Hospital, Tartu, Estonia; Children’s Clinic, Tartu University Hospital, Tartu, Estonia; Alder Hey Children’s NHS Foundation Trust, Liverpool, UK; Research and Development, Liverpool women’s NHS Foundation Trust, Liverpool, UK; Pharmacy Department, Hospital Antoine Beclère, Paris, France; Pharmacy Department, APHP, Hospital Robert Debré, Paris, France; Clinical and Practice Research Group, School of Pharmacy, Queen’s University Belfast, Belfast, UK; Department of Pharmacy, University Hospitals of Leicester, Leicester, UK; Department of Women’s and Children’s Health, Institute of Translational Medicine, Liverpool Women’s NHS Foundation Trust, University of Liverpool, Liverpool, UK

**Keywords:** Neonate, Antibiotics, Beta-lactam, Gentamicin, Vancomycin

## Abstract

**Background:**

Antibiotic dosing in neonates varies between countries and centres, suggesting suboptimal exposures for some neonates. We aimed to describe variations and factors influencing the variability in the dosing of frequently used antibiotics in European NICUs to help define strategies for improvement.

**Methods:**

A sub-analysis of the European Study of Neonatal Exposure to Excipients point prevalence study was undertaken. Demographic data of neonates receiving any antibiotic on the study day within one of three two-week periods from January to June 2012, the dose, dosing interval and route of administration of each prescription were recorded. The British National Formulary for Children (BNFC) and Neofax were used as reference sources. Risk factors for deviations exceeding ±25% of the relevant BNFC dosage recommendation were identified by multivariate logistic regression analysis.

**Results:**

In 89 NICUs from 21 countries, 586 antibiotic prescriptions for 342 infants were reported. The twelve most frequently used antibiotics – gentamicin, penicillin G, ampicillin, vancomycin, amikacin, cefotaxime, ceftazidime, meropenem, amoxicillin, metronidazole, teicoplanin and flucloxacillin – covered 92% of systemic prescriptions. Glycopeptide class, GA <32 weeks, 5^th^ minute Apgar score <5 and geographical region were associated with deviation from the BNFC dosage recommendation. While the doses of penicillins exceeded recommendations, antibiotics with safety concerns followed (gentamicin) or were dosed below (vancomycin) recommendations.

**Conclusions:**

The current lack of compliance with existing dosing recommendations for neonates needs to be overcome through the conduct of well-designed clinical trials with a limited number of antibiotics to define pharmacokinetics/pharmacodynamics, efficacy and safety in this population and by efficient dissemination of the results.

**Electronic supplementary material:**

The online version of this article (doi:10.1186/s12887-015-0359-y) contains supplementary material, which is available to authorized users.

## Background

Recent data suggest that, depending on NICU level, up to 20–40% of admitted neonates receive antibiotic therapy on any particular day, with 30–90% of them exposed to at least one antibiotic during their admission [1-4]. The vast majority of antibiotics are used off-label; data on dosing in this patient group are limited or are generally based on expert opinion or small studies not including all gestational age (GA) groups [2-5]. While some questionnaire-based studies of neonatal antibiotic doses suggest compliance with existing dosing recommendations, others, including unpublished data from the Antimicrobial Resistance and Prescribing in European Children (ARPEC) point prevalence study (PPS), have highlighted large variations in dosing regimens of many commonly used antibiotics in neonates [6-8]. Although therapeutic indications may significantly affect dosing in older children, such variation is not generally expected in neonates. Unexplained dosing variability is a marker of suboptimal patient management meaning that some babies are under- while others are over-dosed. This raises a number of questions about how to deliver optimal dosage of antibiotics to neonates. Is more research needed, or should efforts to disseminate existing evidence be improved?

Extant data do not provide a basis to identify which actions are needed to overcome variability. Previous research efforts to understand which doses of antibiotics are used in neonates have focused on agents with a narrow therapeutic window and/or unfavourable side effect profile or extreme lack of neonatal data, like vancomycin, fluconazole or ciprofloxacin [6,8,9]. Two approaches have been applied, i.e. questionnaire-based studies of policy about dosing regimens and safety data retrieval from the literature [8-13]. Such studies do not allow estimation of actual drug use distribution and are prone to underestimation of variability, as dosing regimens that have been used are not recorded.

In order to improve this situation it is important to understand the causes of the variation. We aimed first, to collect and document details regarding the dosing of antibiotics in European neonatal units and second, to determine whether there are systematic influences on doses which could be the target for interventions to rationalise prescribing.

## Methods

A sub-analysis of the multicentre single day PPS of the European Study of neonatal Exposure to Excipients (ESNEE), detailed elsewhere, was performed [14,15]. The study included 27 European Union countries plus Iceland, Norway, Switzerland and Serbia. General neonatal, intermediate and NICU as well as mixed paediatric and neonatal intensive care units with more than 50% of admissions consisting of neonates were eligible.

For current analysis, all prescriptions for systemic antibiotics in neonates up to 90 days of age, active on the study day morning, chosen by the unit within one of three fixed two-week study periods from January to February; March or May to June, 2012, were retrieved from the ESNEE PPS database. Topical antibiotics, antivirals and antifungals were excluded. Anonymised demographic data including gender, gestational age (GA), birth weight (BW), 1 and 5 minute Apgar score, current body weight (CBW) and postnatal age (PNA) were recorded for each neonate. Postmenstrual age was calculated based on GA and PNA. Due to differences in the pharmacokinetics of antibiotics between term and preterm neonates, but also fast changes occurring over the first weeks of life, dosing recommendations for this age group are based not only on body weight but also GA and/ or PMA. In the dosing analysis further grouping by PMA or PNA was based on the recommendations for the respective antibiotic in the chosen dosing references (Tables 1, 2). Prescription data included active ingredient, route of administration and individual dosing regimen (unit dose and dosing interval) together with prescription start date.

**Table 1:** **The dosing of frequently used beta-lactam antibiotics in European NICUs**

**Drug**	**PNA group**	**No of cases**	**Unit dose (mg/kg/dose)**				**Dosing interval (h)**		
			**Current study; median (quartiles)**	**Current study; range**	**BNFC/Blue book**	**Neofax**	**Current study; median (quartiles)**	**BNFC/the Blue book**	**Neofax**
**Penicillin G**	0–7 days	62	47 (27–53)	10–147	25 (50)*^#^	15–30 (45–60)*	12 (12–12)	12	12
**N = 88**	8–28 days	21	33 (31–50)	25–61	25 (50)*^#^	15–30 (45–60)*	12 (12–12)	8	8–12
	>28 days	5	40 (30–43)	28–43	25 (50)*^#^	15–30 (45–60)*	12 (10–12)	6 (4)	6–8
**Ampicillin**	0–7 days	69	52 (50–78)	24–126	30 (60; max 62.5)*	25–50	12 (12–12)	12	12
**N = 82**	8–21 days	6	52 (49–96)	47–96	30 (60; max 62.5)*	25–50	8 (8–12)	8	8–12
	22–28 days	4	47 (37–86)	34–99	30 (60; max 62.5)*	25–50	12 (8–12)	6	8–12
	>28 days	3	48 (45–69)	45–69	62.5	25–50	12 (8–12)	6	6–8
**Cefotaxime**	0–7 days	16	46 (29–51)	10–54	25 (50)*	50	12 (12–12)	12	12
**N = 32**	8–21 days	12	48 (25–50)	13–53	25 (50)*	50	8 (8–12)	8	8–12
	>21 days	4	47 (16–49)	5–49	25 (50)*	50	8 (8–8)	6–8	8–12
**Ceftazidime**	0–7 days	6	30 (25–41)	22–53	25 (50)*&	30	12 (8–12)	24	12
**N = 20**	8–21 days	5	43 (32–47)	27–49	25 (50)*	30	12 (8–12)	12	8–12
	>21 days	9	36 (30–39)	24–47		30	8 (8–12)	8	8–12
**Meropenem**	0–7 days	10	20 (19–22)	9–39	20 (40)*	20–40*	12 (8–12)	12	12
**N = 20**	8–28 days	10	21 (15–30)	9–43	20 (40)*	20–40*	10 (8–12)	8	8–12

* – the dose in parentheses is recommended for severe infections and/or meningitis.

^#^ – for meningitis 75 mg/kg recommended in the Blue Book.

& – only the higher dose of 50 mg/kg recommended by the Blue Book.

PNA – postnatal age; BNFC – British National Formulary for Children.

The number of prescriptions with accurate dosing data available is shown for each antibiotic. Data are shown as median and quartiles.

**Table 2:** **The dosing of frequently used aminoglycosides and glycopeptides in European NICUs**

**Drug**	**PMA group**	**No of cases**	**Median unit dose (quartiles; mg/kg/dose)**	**Unit dose range (mg/kg/dose)**	**Median dosing interval (quartiles; h)**	**BNFC/the Blue book unit dose (mg/kg/dose)**	**BNFC/the Blue book dose interval (h)**	**Neofax unit dose (mg/kg/dose)**	**Neofax dose interval (h)** ^**c**^
**Gentamicin**	<32 weeks	21	4.6 (4.2–5.0)	3.3–6.6	36 (24–36); 24–48	4–5 (2.5^a^)	36 (18–24^a^)	4–5	36 (24–48)
**N = 139**	≥32 weeks	118	4.4 (3.9–4.8)	1.2–19.4	24 (24–24); 12–36	4–5 (2.5^a^)	24 (12–18^a^)	4	24 (24–36)
**Amikacin** ^**e**^	PNA 0–28 days	34	15.1 (8.8–17.4)	3.1–18.5	24 (24–36); 12–48	15 (7.5^a^)	24 (12)	15–18	24–48
**N = 35**	PNA >28 days	1	7.5	12		15 (7.5^a^)	24 (12)	15	24
**Vancomycin** ^**b**^	<29 weeks	5	9.5	9.0–10.0	21 (11–24); 8–24	15	24	10 (15^d^)	12–18
**N = 40**	29–35 weeks	14	10.0 (9.3–14.3)	4.8–15.6	15 (12–24); 2–48	15	12	10 (15^d^)	8–12
	>35 weeks	21	11.2 (9.7–14.7)	9.0–18.9	12 (8–12); 8–24	15	8	10 (15^d^)	6–12

PMA – postmenstrual age; PNA – postnatal age.

^a^ – multiple daily dosing recommendation regimen is given in parenthesis; for gentamicin multiple daily dosing regimen recommendations are for PMA limits <29 weeks; 29–35 weeks and >35 weeks and have been adjusted accordingly.

^b^ – 9 infants received vancomycin continuous infusion and are excluded from this analysis.

^c^ – different PMA/ PNA groups are applied compared to BNFC, the interval range includes all applicable in the respective PMA/PNA range.

^d^ – meningitis dose.

^e^ – please note, that in contrast to gentamicin and vancomycin dosing based on PMA, current amikacin dosing recommendation is based on PNA.

Because most Summary Product Characteristics (SPC) lack neonatal dosing recommendations [2], the Manual of Childhood Infections (the Blue Book 2010), the British National Formulary for Children (BNFC) 2010–2011 [16,17] and Neofax 2010, 23^rd^ edition [18] were chosen as the guidance documents of drug dosing in neonates, i.e. current editions at the start of the study. BNFC, although country specific by origin, has gained Europe-wide acceptance, as one of the few dosing references, which brings together authoritative, independent guidance on best practice and clinically validated drug information, including that on neonates [2]. The Blue Book and BNFC showed only minimal variations in dosing recommendations. Therefore, apart from the differences, highlighted separately, for the sake of clarity and space data referring to the BNFC is in the focus of results. For uniformity, in dose deviation assessment all prescriptions were translated into CBW adjusted daily doses. The lowest unit dose for the respective PNA or postmenstrual age (PMA) group as appropriate with the longest interval, including once daily (extended interval) dosing regimen for aminoglycosides, recommended in BNFC, was used as the reference (zero line).

### Statistical analysis

Statistical software R (version 2.15.1; © 2012 The R Foundation for Statistical Computing) and Microsoft Excel (version 14.0.7106.5003; © 2010, Microsoft Corporation) were used for analysis. Descriptive statistics are presented as mean and standard deviation (SD) or median and quartiles (IQR) as appropriate. In order to assess influences on prescribing practice, demographic parameters, European region and antibiotic class were investigated for associated risk of deviation from the BNFC daily dose recommendation using univariate logistic regression analysis. A conservative ±25% deviation was accepted to allow for possible dose rounding, except in the case of beta-lactams. For beta-lactam antibiotics the upper limit was set at +125%, as for severe infections and meningitis doubling the dose is recommended and indication of the antibiotic therapy was not collected in the study. Risk factors significant at p < 0.1 in univariate analysis were entered into multiple regression analysis with step-wise removal of the least significant parameters.

The study was approved by the Ethics Committee of the University of Tartu; approval in participating countries was obtained in compliance with national guidelines. The list of ethics committees that approved the study is provided in additional material [See Additional file 1]. None of the ethics committees requested informed consent from parents as all collected data were anonymised and there was no interference with management of patients.

## Results

**Figure 1:**
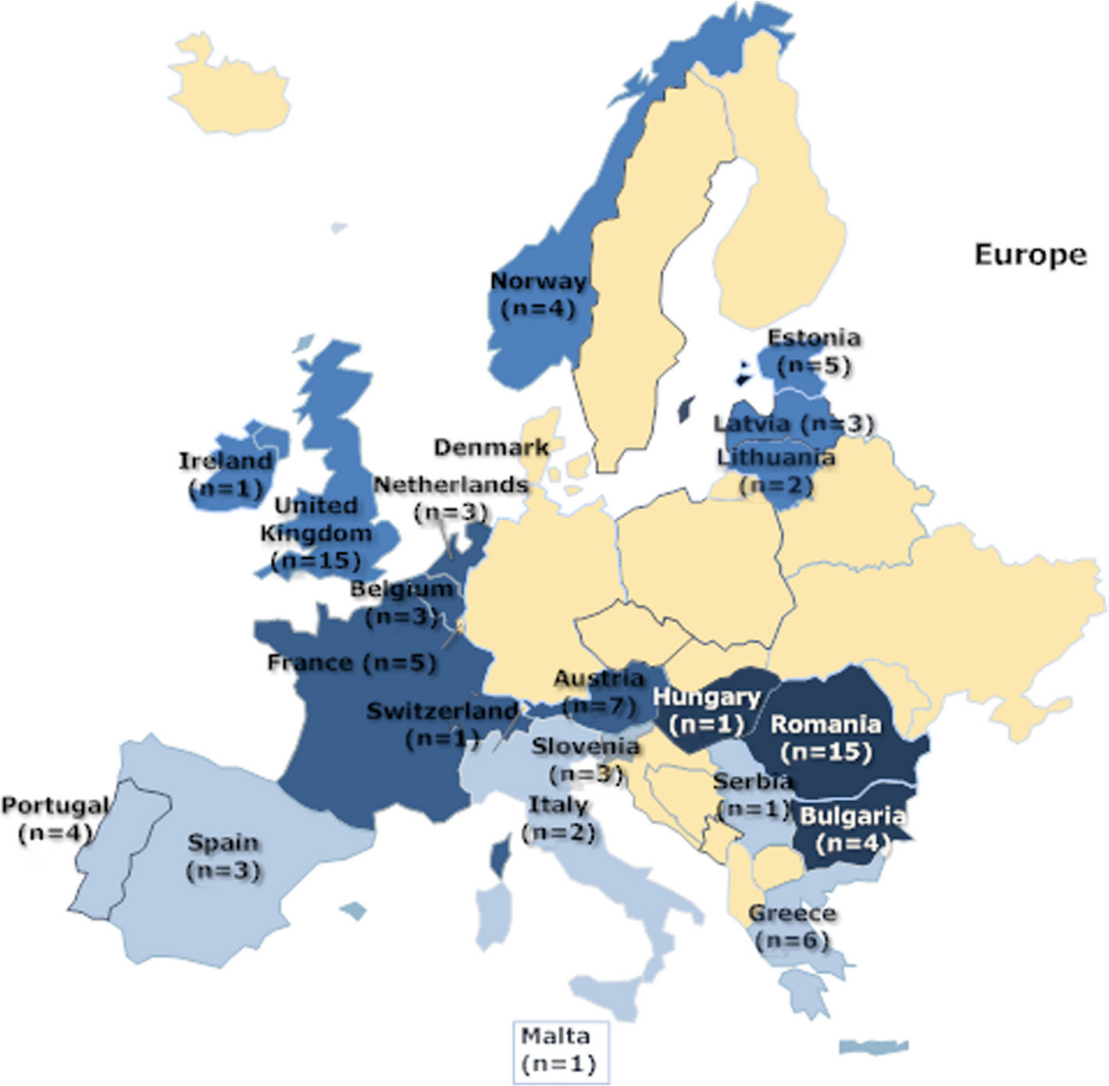
Participating countries by European region (shown in different shades of blue). Number of NICUs participating from each country is shown in parentheses.

Of the 31 invited European countries 21 with 89 NICUs in 73 hospitals participated in the study (Figure 1) [14,19]. Intermediate and 3^rd^ level intensive care units predominated in all participating countries [15]. The median number of neonates reported per country was 45 (quartile range 26.5–65). A total of 2608 prescriptions were reported for 1382 patients, with a mean (SD) BW of 2060 (1032) g and GA of 33 (5) weeks. Among them 342 (25%) patients with mean (SD) BW of 2239 (1075) g and GA of 34 (5) weeks received 586 antibiotic prescriptions (22% of all prescriptions). A full list of antibiotics prescribed to neonates is provided in additional material [see Additional file 2]. The proportion of neonates receiving systemic antibiotics by country ranged from 4% to 78% with a median of 26%. The median number of antibiotic prescriptions per patient was 2 (range 1–5). There were 573 (98%) parenteral and 13 (2%) enteral prescriptions. Three neonates were older than 90 days PNA and were excluded from further analysis.

### Most frequently used antibiotics

A total of 37 different systemic antibiotics were prescribed. Twelve most frequently used active pharmaceutical ingredients – gentamicin, penicillin G, ampicillin, vancomycin, amikacin, cefotaxime, ceftazidime, meropenem, amoxicillin, metronidazole, teicoplanin and flucloxacillin – covered 538, i.e. 92% of the antibiotic prescriptions. Penicillins and aminoglycosides were by far the most frequently prescribed antibiotic classes in all regions, responsible for 212 (36%) and 178 (30%) of 586 prescriptions, respectively. While ampicillin and penicillin G were prescribed at equal frequency in West-European NICUs; in North-European NICUs penicillin G (OR 11.9; 95% CI 5.8–24.4 compared to East- and South-European NICUs) and in East - and South -European NICUs ampicillin (OR 4.3; 95% CI 2.4–7.8 compared to North- and West-Europe) predominated. Gentamicin with 139 prescriptions (78% of all aminoglycoside prescriptions) was the most frequently used aminoglycoside, followed by amikacin. Glycopeptides were prescribed more often in West- and South- compared to North- and East- (OR 2.3; 95% CI 1.4–4.0) and carbapenems in South- and East- compared to North- and West- European NICUs (OR 4.4; 95% CI 1.6–12.1).

Accurate information on the start of prescription were available for 560/586 (96%) of the antibiotic prescriptions. Overall 347 (62%) prescriptions were started within the first three days of life, including over 80% of ampicillin, penicillin G and gentamicin prescriptions. Cefotaxime, amikacin and meropenem had 70% of prescriptions started by the end of the first week of life. In contrast the cumulative number of ceftazidime and vancomycin prescriptions increased steadily over the first month of life. The cumulative proportion of prescriptions for eight most frequently used antibiotic prescriptions by post-natal age is provided in additional material [see Additional file 3].

### Dosing regimen of antibiotics

Antibiotics with 20 or more prescriptions were selected for detailed analysis (n = 8). Deviations of more than 300% of the recommended daily dose were assumed to be erroneous and were excluded. Dosing regimen analysis included 412 prescriptions for five beta-lactam antibiotics (penicillin G, ampicillin, cefotaxime, ceftazidime and meropenem), two aminoglycosides (gentamicin and amikacin) and vancomycin. The median doses as compared to BNFC 2010–2011 Ed [17] and NeoFax (23^rd^ Edition, 2010) [18] recommendations by drug class and PNA/PMA band as appropriate, are shown in Table 1 (beta-lactams) and Table 2 (aminoglycosides and vancomycin). Lower doses of penicillin G and ampicillin and higher doses of cefotaxime were recommended by Neofax compared to BNFC.

For penicillin G the median unit dose was close to the BNFC recommendation for severe infections in the youngest and to that of the Neofax in older PNA bands (Table 1). Ampicillin median unit doses were at the high end of the Neofax reference in all PNA bands. No reduction in dosing interval with increasing PNA was seen. The median dose for cefotaxime equaled the BNFC reference for meningitis and severe infections. The dosing intervals, similar in both cited references, were followed with the only exception of BNFC recommendation of 24 h for ceftazidime during the first week of life. Meropenem median dose was equal to the routine recommendation, dosing intervals in the PNA > 7 days group exceeding the BNFC but in line with the Neofax reference.

For gentamicin and vancomycin different PMA groups with generally similar unit doses for gentamicin and higher doses of vancomycin in the BNFC, were suggested (Table 2). Amikacin dosing recommendations were based on PNA group. Gentamicin median unit doses were within the reference range but in 34 cases (24%) dosing intervals exceeded the longest recommended (once daily regimen). The BNFC recommended multiple daily dosing interval was used in 7 (33%) and 12 (10%) neonates in the <32 weeks and ≥32 weeks PMA group, respectively. Vancomycin unit doses remained below the reference range in three quarters of cases with shorter than recommended intervals used in only a quarter of cases.

### Variability of daily doses

**Figure 2:**
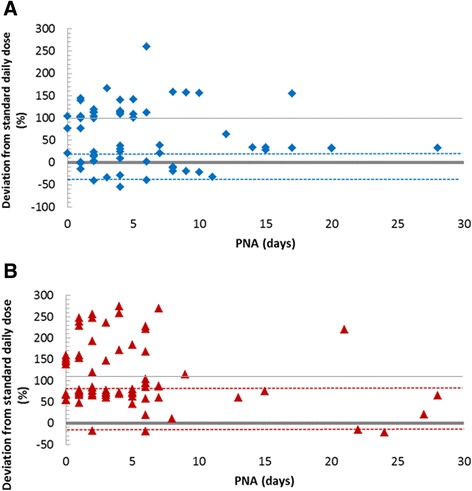
Variation in the dosing (mg/kg/day) of penicillin G (panel **A**) and ampicillin (panel **B**) in comparison to British National Formulary for Children 2010–2011 recommendations. The minimum accepted unit dose (mg/kg/dose) and dosing interval for the respective PNA group was used for the calculation of the reference daily dose (zero line). Maximum BNFC recommended daily dose is shown in light grey line. Dotted coloured lines depict minimum and maximum Neofax 2010 dosing recommendation. Each dot represents a different patient.

**Figure 3:**
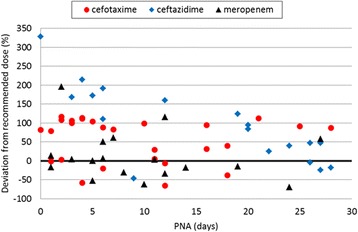
Variation in the dosing (mg/kg/day) of cefotaxime, ceftazidime and meropenem in comparison to British National Formulary for Children 2010–2011 recommendations. The minimum accepted unit dose (mg/kg/dose) and dosing interval for the respective PNA group was used for the calculation of the reference daily dose (zero line).

**Figure 4:**
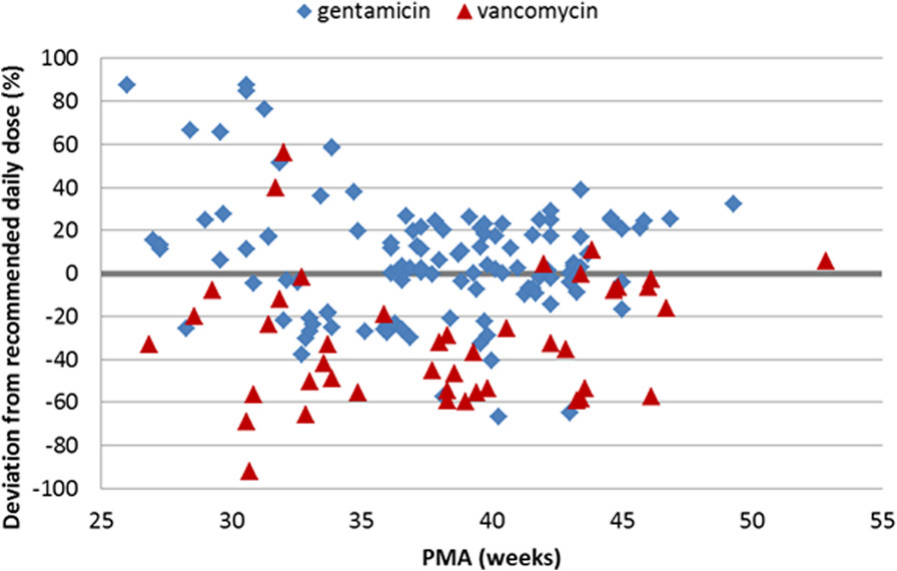
Variation in the dosing of gentamicin and vancomycin (mg/kg/day) in comparison to British National Formulary for Children 2010–2011 recommendations. The minimum accepted unit dose (mg/kg/dose) and dosing interval for the respective PNA group was used for the calculation of the reference daily dose for all drugs (zero line). For gentamicin the once daily (extended interval) dosing regimen was applied.

Deviations from the BNFC reference daily dose for non-severe infections are depicted in Figure 2 and Figure 3 for beta-lactams and in Figure 4 for gentamicin and vancomycin. In general, penicillins were given at higher doses than recommended for non-severe infections. The highest recommendation for severe infections like meningitis, osteomyelitis and endocarditis (calculated as +125% of routine dose) was exceeded in 26/82 (32%) of ampicillin cases and 10/69 (14%) of those involving penicillin G. For cefotaxime 18/30 (60%) prescriptions were within the ±25% of the highest recommended dose. Ceftazidime doses exceeded the maximum for severe infections during the first 7 days but not thereafter (Figure 3). Daily doses remained below the reference dose in only 12% of beta-lactam prescriptions, most frequently for meropenem (6/20; 30%), followed by cefotaxime (3/30; 10%). The lowest doses of penicillins were in line with the Neofax recommendation (dotted lines on Figure 2).

Gentamicin daily doses were above or below the ±25% range of the lowest daily recommendation in 13 (62%) and 21 (18%) cases in the <32 weeks and ≥32 weeks PMA groups, respectively. Higher than recommended daily dosing was the result of shorter intervals rather than higher unit doses. For vancomycin the majority of doses (40/48; 83%) remained below the BNFC lowest reference independent of the PMA group and variability was highest at <36 weeks of PMA.

In univariate logistic regression analysis BW ≤1500 g; GA ≤32 weeks, PMA <29 weeks; 1^st^ minute Apgar score ≤3; 5^th^ minute Apgar score ≤7, treatment in West-European NICU and glycopeptide class prescription were associated with higher risk of deviation from the BNFC recommended dose. On the other hand any beta-lactam, cephalosporin or aminoglycoside prescription and treatment in North-European NICU had a lower risk of dose deviation (data not shown).

The results of multivariable logistic regression analysis are presented in Table 3. Glycopeptide class prescription was the strongest risk factor of major dose deviation, followed by treatment in West-European NICU, 5^th^ minute Apgar score <5 and gestational age <32 weeks. Treatment in a North-European NICU was the only factor independently associated with a lower likelihood of major deviations from BNFC dosing recommendation.

**Table 3:** **Risk factors of AB dose deviation; multiple regression analysis**

**Risk factor**	**OR**	**95% CI**
**Glycopeptide prescription**	6.9	3.1–15.4
**West-Europe**	2.2	1.04–4.6
**5** ^**th**^ **minute Apgar score <8**	1.8	1.1–3.0
**GA <32weeks**	1.7	1.02–2.8
**North-Europe**	0.6	0.3–0.99

## Discussion

In a Europe-wide study looking at the antibiotic dosing in neonates at the individual level, we have demonstrated extremely wide and non-random variations in dosing of the most frequently used antibiotics. In general, antibiotics known to be well tolerated (e.g. penicillins and cephalosporins) were given in higher doses than the reference range. Those with safety concerns were given in line with (e.g. gentamicin), or at lower than the reference doses (e.g. vancomycin). Deviations from reference values tended to be greater during the first 7 days of life for beta-lactams and in less mature babies (PMA < 32/36 weeks) for gentamicin and vancomycin, compared to other PNA/PMA groups. Although antibiotics are by far the most commonly used medicines in neonates [2,3] more than 90% of the prescriptions in our study included only 12 different antibiotics suggesting that defining appropriate dosing recommendation for a relatively small number of agents would resolve the issue of inappropriate/inadequate dosing for most hospitalised neonates in Europe.

The reasons for the high variability in dosing of antibiotics in neonates are likely multiple. First, high quality data relevant to dose selection is lacking for many antibiotics used in neonates [20]. Current guidelines are based mainly on expert opinion and small studies rather than on well conducted clinical trials, leading to wide inter-centre variability in dosing recommendations [2,21,22]. In a recent study of 56 French NICUs 444 dosing regimens were reported for 41 antibiotics [22]. Uncertainty about the dosing and safety as well as need for additional studies of efficacy were among the most frequently mentioned factors influencing decisions in a Europe-wide study of antifungal prophylaxis in neonates [13]. Safety concerns as a basis for dosing variations are well reflected by the different pattern seen for antibiotics with wide vs narrow therapeutic window in our study. Similarly, a recent study looking at dosing of antifungals (generally associated with poor tolerance and unfavourable side effect profile) in European children, found under-dosing in about half of the cases [23].

Additional difficulties are posed by changes in the PK of antibiotics over the first few weeks of life, a factor that requires appropriate dose adjustments [24]. The best compliance with existing dose recommendations was seen for drugs like cephalosporins and meropenem, with more recent, adequate PK data resulting in similar dose recommendations in both reviewed references [25-30]. In contrast, the PNA-specific rule in the BNFC for ceftazidime (once daily dosing over the first week of life followed by twice daily thereafter) was not followed, despite clear evidence from PK studies [31].

Other reasons of the high variability in dosing include problems in adoption and dissemination of evidence based knowledge.The former is reflected by penicillin G dosing recommendations contradicting recent PK studies [32-34]. Evidence of poor dissemination of changing recommendations and the relative conservatism of the clinical community is provided by the examples of aminoglycosides and vancomycin. The once daily or extended interval dosing of aminoglycosides, supported by PK and clinical studies including safety assessment in neonates [35-37], appears to be not yet completely accepted after two decades of intense debate. Multiple daily dosing with less than a 24h interval was used in 14% of cases in our study. Vancomycin was associated with the highest likelihood of major dose deviations, reflecting difficulties in adopting more recent and probably more controversial dosing recommendations of an agent known to have significant side effects [38]. There is clearly a need for improved dissemination of evidence-based guidelines in European neonatal units via professional societies or regulatory initiatives, where appropriate.

Clinicians can adapt to well-founded guidelines that are adequately disseminated. In our study this is reflected by differences between European regions. Western Europe, likely relying on dosing references other than the BNFC alone, was associated with the highest likelihood of deviation from the BNFC reference. In contrast, Northern Europe, predominately represented by UK NICUs, had the best compliance with BNFC recommendations.

The main limitation of the study is the lack of data on the indications for which antibiotics were prescribed. The majority of antibiotics are prescribed to neonates for suspected rather than proven infection [39]. In our study about 2/3 of antibiotics were prescribed within the first three days of life. Only 3–8% of such neonates have been found to develop proven sepsis [39-41]. Infections like meningitis, osteomyelitis or endocarditis requiring specific dosing of beta-lactam antibiotics, are extremely rare in neonates [17,18,39]. The reported incidences range from 0.21 and 0.12 per 1000 live births for meningitis and osteomyelitis, respectively [42,43] to 0.7 per 1000 Special Care Nursery admissions in the case of endocarditis [44,45]. Therefore, the wide dosing variations seen in our study cannot be explained by treatment indications. Furthermore, accepting the dose recommended for severe infections in all cases, as done in our analysis, reflects the most conservative approach. BNFC was used as the dosing reference in this analysis, while other sources may have been used in some centres. However, this fact does not undermine our main finding of the high variability in the dosing of most frequently used antibiotics in neonates. Neither clinical studies nor epidemiological data provide any rational explanation. In addition, therapeutic drug monitoring (TDM) data may have affected vancomycin and aminoglycoside dosing in the study. Even assuming TDM results to be the underlying reason for all dose deviations supports our thesis that the precision/value of current dosing recommendations needs to be or is being questioned by clinicians. As the data were collected in a large multi-national setting with no data monitoring and queries applied, extreme deviations were excluded from analysis.

## Conclusions

In conclusion, the prescribing practice of antibiotics in European neonatal units is highly variable but is not random. There are some systematic influences on dose being selected. These influences stem from demographic and geographic variables and from the class of antibiotics used. Clinicians’ and experts’ distrust of newer dosing recommendations needs to be overcome by adequate clinical trials to support PK/PD, efficacy and safety. In addition, new data should be critically reviewed and SPCs, together with internationally accepted guidelines, updated accordingly. The ultimate goal for the paediatric community would be to have evidence based dosing recommendations for the most commonly used antibiotics and for these to be disseminated effectively. Our study suggests a strategy to optimise antibiotic prescribing for European neonates. Firstly, improved dissemination strategies are urgently needed for antibiotics with clear evidence to support a dosage regimen, e.g. aminoglycosides and cephalosporins. Secondly, other antibiotics need an improved evidence base that could be disseminated using the knowledge translation strategies that have been applied for aminoglycosides.

## Additional files

Additional file 1:
**List of ethics committees that approved the European Study of Neonatal Exposure to Excipients point prevalence study.**
Additional file 2:
**List of systemic antibiotics prescibed to neonates participating in the point prevalence study of the European Study of Neonatal Exposure to Excipients.**
Additional file 3:
**Cumulative proportion of prescriptions for eight most frequently used antibiotic prescriptions by post-natal age.**

## Abbreviations

ARPEC: The antimicrobial resistance and prescribing in European children
BNFC: The British National Formulary for Children (BNFC)
BW: Birth weight
CBW: Current body weight
ESNEE: European study of neonatal exposure to excipients
GA: Gestational age
NICU: Neonatal intensive care unit
PMA: Postmenstrual age
PNA: Postnatal age
PPS: Point prevalence study
SPC: Summary product characteristics
