# Supplementary material for: High variability in the dosing of commonly used antibiotics revealed by a Europe-wide point prevalence study: implications for research and dissemination
Source: BMC Pediatr. 2015 Apr 16;15:41. doi: 10.1186/s12887-015-0359-y (PMC4407781; doi:10.1186/s12887-015-0359-y)
Supplement: Additional file 1: — List of ethics committees that approved the European Study of Neonatal Exposure to Excipients point prevalence study. [file 12887_2015_359_MOESM1_ESM.pdf]

Ethics committees that approved the European Study of Neonatal Exposure to Excipients point prevalence study

Universitair Ziekenhuis Gent, Comissie voor Mediche Ethiek, Belgium

Scientific Committee of each participating hospital in Greece

Research Ethics Committee of the University of Tartu, Estonia

Ethics Committee of the Semmelweis University Clinic, Hungary

Ethics Committee of the National Maternity Hospital, Dublin Ireland

Comitato di Bioetica della Provincia Romana del FBF, Italy

Kauno Regioninis Biomedicininiu Tyrimu Etikos Komitetas, Lithuania

University of Malta Research Ethics Committee –UREC, Malta

Institutional Review Board of the Erasmus MC, the Netherlands

Komisija Republike Slovenije Medicinsko Etiko, Slovenia

Comite Etico de Investigacion Clinica de EUSCADI (CEIC-E), Spain

Kantonale Ethikkomission Zürich, Switzerland

NRES Committee East of England – Cambridge Central, National Ethics Service, UK; NHS/HSC

Ethics Committee approval was not required in Austria, France, Portugal, Latvia, Serbia and Bulgaria.
