# Supplementary material for: High variability in the dosing of commonly used antibiotics revealed by a Europe-wide point prevalence study: implications for research and dissemination
Source: BMC Pediatr. 2015 Apr 16;15:41. doi: 10.1186/s12887-015-0359-y (PMC4407781; doi:10.1186/s12887-015-0359-y)
Supplement: Additional file 2: — List of systemic antibiotics prescibed to neonates participating in the point prevalence study of the European Study of Neonatal Exposure to Excipients. [file 12887_2015_359_MOESM2_ESM.pdf]

List of systemic antibiotics prescibed to neonates participating in the point prevalence study of the European Study of Neonatal Exposure to Excipients.

1	Amikacin
2	Amoxicillin
3	Amoxicillin	Clavulanic acid
4	Ampicillin
5	Ampicillin	Sulbactam
6	Azithromycin
7	Benzylpenicillin
8	Cefalexin
9	Cefepime
10	Cefotaxime
11	Cefoxitin
12	Ceftazidime
13	Ceftriaxone
14	Cefuroxime
15	Ciprofloxacin
16	Clarithromycin
17	Clindamycin
18	Colistimethate
19	Colistin
20	Ertapenem
21	Flucloxacillin
22	Fosfomycin
23	Gentamicin
24	Josamycin
25	Kloksacillin
26	Lincomycin
27	Meropenem
28	Metronidazole
29	Netilmicin
30	Oxacillin
31	Piperacillin	Tazobactam
32	Spiramycin
33	Teicoplanin
34	Trimethoprim
35	Vancomycin
