# Supplementary figures and images for: High variability in the dosing of commonly used antibiotics revealed by a Europe-wide point prevalence study: implications for research and dissemination
Source: BMC Pediatr. 2015 Apr 16;15:41. doi: 10.1186/s12887-015-0359-y (PMC4407781; doi:10.1186/s12887-015-0359-y)

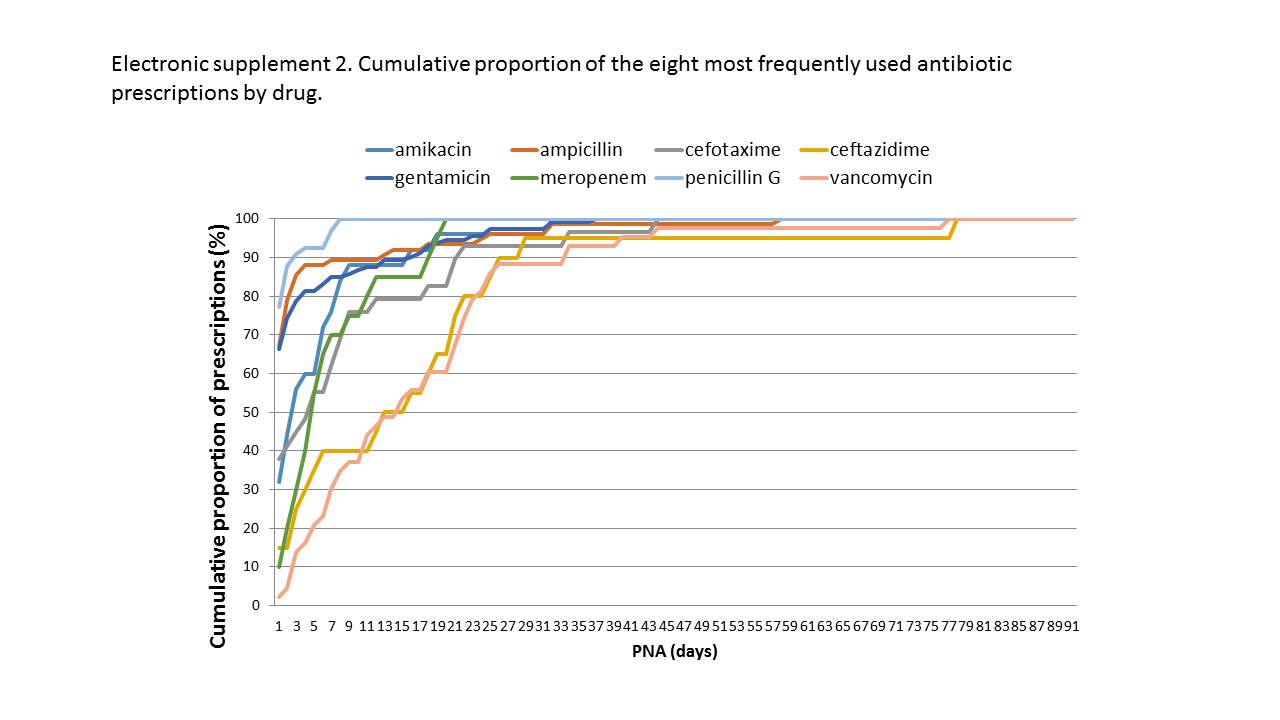

Supplement: Additional file 3: — Cumulative proportion of prescriptions for eight most frequently used antibiotic prescriptions by post-natal age. [file 12887_2015_359_MOESM3_ESM.tiff]
